# Ageing in general practice (AGP) trial: a cluster randomised trial to examine the effectiveness of peer education on GP diagnostic assessment and management of dementia

**DOI:** 10.1186/1471-2296-13-12

**Published:** 2012-03-07

**Authors:** Constance D Pond, Henry Brodaty, Nigel P Stocks, Jane Gunn, John Marley, Peter Disler, Parker Magin, Nerida Paterson, Graeme Horton, Susan Goode, Bronwen Paine, Karen E Mate

**Affiliations:** 1 School of Medicine and Population Health, University of Newcastle, Newcastle, Australia 2308; 2 School of Psychiatry, University of New South Wales, Sydney, Australia 2052; 3 School of Population Health and Clinical Practice, The University of Adelaide, Adelaide, Australia 5005; 4 Department of General Practice, University of Melbourne, Melbourne, Australia 3010; 5 School of Rural Health, Monash University, Melbourne, Australia 3800; 6 School of Biomedical Science & Pharmacy, University of Newcastle, Newcastle, Australia 2308

## Abstract

**Background:**

Dementia is increasing in prevalence as the population ages. An earlier rather than later diagnosis allows persons with dementia and their families to plan ahead and access appropriate management. However, most diagnoses are made by general practitioners (GPs) later in the course of the disease and are associated with management that is poorly adherent to recommended guidelines. This trial examines the effectiveness of a peer led dementia educational intervention for GPs.

**Methods:**

The study is a cluster randomised trial, conducted across three states and five sites. All GPs will complete an audit of their consenting patients aged 75 years or more at three time points - baseline, 12 and 24 months. GPs allocated to the intervention group will receive two educational sessions from a peer GP or nurse, and will administer the GPCOG to consenting patients at baseline and 12 months. The first education session will provide information about dementia and the second will provide individualised feedback on audit results. GPs in the waitlist group will receive the RACGP Guidelines by post following the 12 month audit

Outcomes: Primary outcomes are carer and consumer quality of life and depression. Secondary outcomes include: rates of GP identification of dementia compared to a more detailed gold standard assessment conducted in the patient's home; GP identification of differential diagnoses including reversible causes of cognitive impairment; and GP referral to specialists, Alzheimers' Australia and support services. A "case finding" and a "screening" group will be compared and the psychometrics of the GPCOG will be examined.

Sample size: Approximately 2,000 subjects aged 75 years and over will be recruited through approximately 160 GPs, to yield approximately 200 subjects with dementia (reducing to 168 by 24 months).

**Discussion:**

The trial outlined in this paper has been peer reviewed and supported by the Australian National Health and Medical Research Council. At the time of submission of this paper 2,034 subjects have been recruited and the intervention delivered to 114 GPs.

**Trial registration:**

Australia and New Zealand Clinical Trials Register (ANZCTR): ACTRN12607000117415.

## Background

### Importance of dementia as an issue

Dementia is an increasingly common condition largely due to increasing longevity of the population in Australia, and other countries. Approximately 1 in 5 Australians over the age of 80 have some form of dementia [1]. By 2050 the number of adults 65 to 84 years with dementia is expected to double and the number of people 85 years or older will more than quadruple [2]. Consequently, the number of people living with dementia is anticipated to rise rapidly. It is forecast that almost 1 million Australians will suffer from dementia by 2050, up from 250,000 in 2009 [1].

Dementia is associated with substantial economic, social and emotional burden. The disease is costly in terms of the need for support services whether sufferers live at home or in residential aged care, as well as in terms of lost productivity for carers and people living with dementia [3]. The *2010 Intergenerational Report *estimates that Australian Government spending on aged care will increase from 0.8 per cent of GDP in 2010 to 1.8 per cent by 2050 [2]. In addition to the effect of dementia on the public and private purse, the emotional suffering both of those with the disease and of their families should not be overlooked.

### The role of the GP in identifying and managing dementia

General practitioners (GPs) appear well positioned as the first point of contact for older persons seeking health care, including those living with dementia. In the decade between the 2000 and 2010, the number of patients aged under 45 years seen annually by GPs increased by 190,000, whereas the number of patients aged 45 years and over increased by 16 million [4]. Twenty-eight percent of the 98,800 encounters recorded by the annual *Bettering the Evaluation And Care of Health *(BEACH) survey of GP consultations from April 2009 to March 2010 were with patients aged 65 and over [5].

General practitioners often make the diagnosis of dementia some considerable time into the course of the disease [6,7]. However, earlier recognition of symptoms can allow reversible causes of cognitive decline to be addressed and identification and management of co-morbidities (e.g. depression). An early diagnosis also allows patients time to plan for the future (e.g. prepare wills, appoint enduring powers of attorney and guardians, prepare advance directives) while they are still competent to do so. Referral can be made to appropriate support services and diagnostic centres for further assessment and/or diagnostic confirmation. Anti-dementia medication that may slow the course of cognitive decline may be trialled. Education for patients and families, along with peer services and support groups, may help them understand and cope with the challenging symptoms of dementia. The support role is particularly stressful [8] and such assistance might reduce support person morbidity such as depression, and prepare them for maintaining people with dementia at home longer [9].

General practitioners often fail to identify dementia early in the course of the disease and also adhere poorly to published dementia guidelines [10,11]. They often fail to exclude other possible causes of cognitive impairment, including physical illnesses, medications and depression [10]; are slow to communicate the diagnosis to patients and carers [12,13]; and fail to refer people on to services, support groups, memory clinics or other specialist resources [10].

### Addressing barriers to diagnosis and guideline adherence

Both qualitative and quantitative research suggests a range of reasons for late diagnosis and otherwise poor adherence to guidelines by GPs [14]. The barriers specifically addressed in this study are the limited time for consultation [15], lack of relevant knowledge [16] and attitudinal factors [13,17]. The GPCOG is a brief screening test developed and refined by team members to address the time constraints in general practice consultations [18]. The Australian *Care of Patients with Dementia in General Practice Guidelines *[19] were developed and trialled in consultation with GPs to assist them in the management of dementia. These guidelines form the basis for the education used in this study. The reluctance amongst GPs to diagnose a disease which lacks a known cure and causes immense suffering to those who have it and their families [13,17] is also addressed in the educational intervention.

### Screening vs case finding as a means of identifying dementia

In general, medical advisory groups and policy makers recommend case finding (further testing only for those who are perceived by the GP as having symptoms suggesting possible dementia) rather than broadly screening persons over a certain age for dementia [20,21]. It is thought that screening in this low prevalence population would result in false positives, causing distress to the patient and their support person, as well as being a burden to the healthcare system. Other guidelines have withheld judgment as they claim insufficient evidence for or against screening [21,22]. This study will compare screening and case finding approaches to identifying dementia.

### Rationale for the educational intervention

This trial addresses the problems outlined above with an interactive educational intervention for GPs, which is described in detail below. The education includes visits by a peer health professional educator and an audit with feedback. The small positive effects of educational meetings [23] can be improved if they are interactive [24]; outreach visits have shown promise in modifying the behaviour of professionals [25]; and audit and feedback has been shown to result in modest improvements in outcomes [23,26].

### Aims of the study

1. Compared to a GP group which does not receive peer delivered education, to examine whether training in and use of a dementia screening instrument and relevant information (see *The Intervention *section below) improves:

i. GP identification (diagnosis) of dementia

ii. Distinction by GPs between dementia and other diseases, including depression (hereafter termed "differential diagnosis")

iii. GP elimination of reversible causes of cognitive impairment

iv. Active management of dementia by GPs as evidenced by increased referral to appropriate services, to Alzheimer's Australia and/or to medical specialists for memory problems where indicated.

Outcomes for consumers and their support people in terms of quality of life (primary outcome), depression (primary outcome), satisfaction with care, and access to Alzheimer's Australia, appropriate services or specialists when indicated.

2. To determine whether a screening or a case finding approach to dementia results in:

i. better outcomes for people with dementia and their carers

ii. a more acceptable process for consumers, support people and GPs

3. o test the psychometric properties of the GPCOG including inter-rater reliability, test-retest reliability and comparison with the MMSE..

## Methods/Design

### Study Design

**Figure 1:**
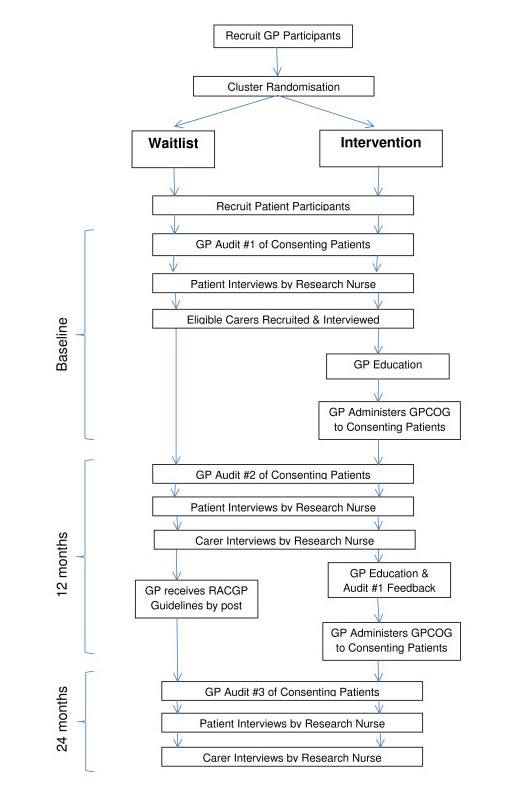
**Summary of recruitment, randomization and study design**.

The trial is a cluster randomised study (summarised in Figure 1). Practices allocated to the Intervention group will receive two dementia related educational detailing visits from a peer GP or a nurse, and complete three audits with feedback. Waitlist practices will complete three audits without feedback and will be mailed RACGP Dementia Guidelines (including the GPCOG) at 12 months.

### Setting

Recruitment will occur across 5 sites: Newcastle (NSW), Sydney (NSW), Melbourne (Victoria), Adelaide (South Australia) and Bendigo (Victoria). Sydney, Melbourne and Adelaide are state capital cities of more than a million people each, Newcastle is a regional centre of around 300,000 people and Bendigo is a smaller centre of just under 100,000 people with some smaller surrounding towns.

### Recruitment of participants

#### GPs

With the exception of Bendigo (which will also include nearby towns as noted above) each site will generate a list of possible GP practices within 30 km of the site headquarters based on lists provided by the local Division of General Practice. Practices will be allocated a random approach order, and will be contacted via phone initially. GPs who express an interest in participation will be visited in order for the study to be explained. Basic demographics will be collected at this visit, from those GPs that agree to participate. It is not expected that all the GPs in a practice will participate. When one or more GPs in a practice consent, the practice as a whole will be allocated to either the intervention or waitlist group. Randomisation will occur at a practice level in order to avoid contamination from patients visiting several GPs who had been randomised to different arms within a single practice.

Randomisation will follow procedures outlined in the CONSORT statement [27]. Practices will be allocated in a ratio of 2:1 to the Intervention or Waitlist group, using an allocation schedule provided independent of the study team, by the Centre for Epidemiology and Biostatistics at the University of Newcastle. The randomisation code has been developed using a computer random number generator to select random permuted blocks. Block lengths of 3 or 6 will be varied randomly. Practices will be stratified by site, and by size of practice as either standard or large (large = more than 7 GPs working in the practice). The intervention group will be kept at twice the size of the waitlist group in order to have sufficient power to compare the effects administering the GPCOG on case finding versus screening patients (see explanation below).

Inclusion criteria for GPs/practices

• Located within 30 kilometres of recruitment centre (with the exception of Bendigo, a rural town)

• Consent to participate in study

• Have patients on a computerised database

• Have patients aged 75 years and over and living in the community

• Consent to being randomised to either intervention waitlist group.

Exclusion criteria for GPs/practices

• Involved in project development

• Does not meet inclusion criteria

### Patients and support persons

Patients will be recruited by a mail out from each consenting GP to all eligible patients on their database. Patient participants will return their consent in reply paid envelopes to the study centre. Patients will be interviewed by study personnel blind to the group allocation of the patient and their GP. For patients scoring in the dementia range on the CAMCOG instrument (described below), the interviewer will request that they nominate a support person, if available, to take part in the study. Nominated support people will be given a consent form and information sheet for recruitment into the study.

Inclusion criteria for patients:

• Aged 75 years or over

• Listed on participating GPs' databases

• Visited recruited GPs within the last 24 months

• Consent to a home visit or surgery visit by project staff

• Speak and understand English

Inclusion criteria for support persons:

• Identified as a carer or support person by a patient scoring in dementia range on CAMCOG instrument

• Prior consent from the person with dementia for his/her carer to participate in the study

• Speak and understand English

• Consent to a home visit or surgery visit by the project staff.

Exclusion criteria for patients:

• Parkinson's disease, multiple sclerosis, motor neuron disease or central nervous system inflammation

• Psychotic symptoms prior to recruitment

• Developmental disability

• Insufficient English to complete a psychometric assessment (judged by interviewer)

• Progressive malignancy

• Substance abuse

• Deemed too sick to complete study by the GP

• Lives in a residential aged care facility at entry to the study

• Does not meet inclusion criteria

• Valid and informed consent cannot be obtained from the person involved and they do not have a person responsible for them.

Exclusion criteria for support persons:

• Insufficient English to complete testing (judged by the interviewer)

• Non-consenting patient-participant

• Does not consent to a home visit or surgery visit by the project staff.

• Too unwell to participate

• De not meet inclusion criteria.

### Case finding and screening

"Case finding" patients will be identified during the patient interview using the following 4 questions:

1. Do you have any complaints about your memory?

2. Have you mentioned these to you GP?

3. If not, have you been intending to tell your GP?

4. Have you sought treatment or taken any remedies such as herbal medications or vitamins specifically for you memory? Please give details.

If questions 2, 3 or 4 are answered in the affirmative, the patient will be considered as a "case finding" patient. Patients identified as having possible or probable dementia on the GP baseline audit will also be considered as a "case finding" patient. All other patients will be identified as "screening" patients - that is, patients who would not receive a cognitive function test unless the GP decided to screen all patients aged over a certain age.

### The intervention

All GPs will audit their consenting patients for dementia, and complete a supplementary audit indicating diagnostic evaluation and management for patients they consider to have dementia. This will occur at three time points during the study: baseline, 12 months and 24 months. Intervention GPs will receive education as described below. The rationale for the educational intervention is described in the *Background *section above.

#### Intervention GPs

Intervention GPs will participate in two visits at their surgery with a peer medical or nurse educator. At the first visit (after completion of the baseline audit), GPs will be taught how to use the GPCOG screening instrument (described below), and will be given information about dementia diagnosis, diagnostic workup and management according to strategies incorporated in the RACGP Consensus Guidelines for the care of Patients with Early Dementia (hereafter called the RACGP Dementia Guidelines) [19]. Negative attitudes about making a dementia diagnosis will be explored and addressed. With the GPs' consent, these sessions will be recorded for qualitative analysis. The structural issue of lack of time in the GP consultation will be addressed by teaching the GPs to use a brief screening instrument and by discussing potential methods of obtaining assistance from the practice nurse. A business case regarding the cost effectiveness of dementia assessment will also be presented. All intervention GPs will be provided with support materials and the RACGP Dementia Guidelines at this visit. These GPs will receive a second detailing visit after completion of their 12 month audit. At the second visit, intervention GPs will receive written and verbal feedback about their baseline audit in the light of individual patient results from the home assessment.

As the study is being conducted across five sites, a number of different peer educators will be used. All educators will undergo a training program either face-to-face or via teleconference to familiarise them with the educational package developed for intervention GPs. The package consists of a slide presentation that will be delivered by educators via laptop computer, and hard-copy support materials that will be supplied to the GPs.

#### Waitlist GPs

Waitlist GPs will complete the audits as per Figure 1. No feedback will be given to waitlist GPs during the course of the study. They will be mailed the RACGP Dementia Guidelines after completion of the 12 month audit. Apart from administrative phone calls to obtain the audits, waitlist GPs will have no other contact with the study team.

### Research instruments

The following instruments will be used be used as part of the patient and/or carer assessments:

#### The GPCOG

The GPCOG [18,28] is a simple dementia-screening instrument, translated into a number of languages and is freely available on the internet http://www.gpcog.com.au. The refined version of the GPCOG has not been trialed in a large random sample of GP patients. It is considered superior to other screening instruments such as the MMSE [29] because of its brevity, psychometric properties and its use of informant report in borderline cases [28].

#### The CAMCOG

The CAMCOG is a subsection of the CAMDEX instrument, which has been validated in the UK [30]. The CAMCOG has the MMSE embedded in it and, thus, allows comparison between the GPCOG and the MMSE. The CAMCOG was used in our previous study to validate the original version of the GPCOG [18].

#### The Geriatric Depression Scale

The Geriatric Depression Scale (GDS) has been well validated and widely used in Primary Health Care research [31]. The GDS-15 will be used to limit the time imposed on participants.

#### The WHOQOL-BREF

The WHOQOL-BREF is a widely used quality of life instrument [32], which has been validated for use in the Australian population [33].

#### The Beck Depression Inventory

The Beck Depression Inventory [34] has been widely used in the general population to screen for depression.

#### The General Practice Assessment Questionnaire

The General Practice Assessment Questionnaire (GPAQ) will be used to ascertain the patients' support persons' satisfaction with their care from their GP over the previous 12 months. The GPAQ is very widely used in the UK as part of the GP quality outcomes framework, and has been shown to be acceptable, reliable and have an interpretable factor structure [35].

#### Activities of Daily Living (ADL)

The Lawton and Brody ADL Questionnaire [36] will be used to measure the functional abilities of patients who score in the dementia range on the CAMCOG instrument. This 13-item questionnaire designed for use in elderly patients with neurological disorders, measures both instrumental (eg ability to manage finances, medications) and basic (eg toileting, grooming) ADL. It will be completed by the support person (if available) or the patient.

### Data collection

#### Patient assessment

The research nurse will use a laptop computer to collect demographic data from all consenting patients and to administer the GPGOG, CAMCOG, GDS, WHOQOL-BREF, GPAQ and ADL (for patients in CAMCOG dementia range only). Patients will be asked about any memory-related services accessed, referral to Alzheimer's Australia, and referral to a specialist for memory problems. The research nurse will compile a list of all prescription and over the counter medications currently used by the patient, so that medication can be excluded as a cause of cognitive impairment. Complementary and alternative medicines will be included. In addition the nurse will perform a "75 + Health Assessment" rebateable under the Australian Medicare system on eligible patients where this has been requested by the GP. These data will not be used by the study, but are returned to the GP for his or her use. This part of the assessment is a provided as a service to the GP. Brief questions used in our previous research will be used to measure the acceptability of the interview process. We will ask patients to send answers back in a reply-paid envelope, to avoid bias due to the presence of the nurse. At the completion of the assessment the nurse will hand the patient a sealed envelope. In the case of patients in the intervention group, it will direct the patient to obtain an appointment with his/her GP as soon as possible to follow up on the 75+ Health Assessment and to have the GPCOG re-administered. In the case of patients in the waitlist group, it will suggest a follow up for the 75 Plus Health Assessment only. Patients will be reassessed using the same instruments at 12 and 24 months after baseline interviews.

#### Support person assessment

The research nurse will use a laptop computer to collect demographic data from consenting support persons and to administer the WHOQOL-BREF, BDI and GPAQ.

Information regarding services accessed and acceptability of the interview process will be collected as described above for patients. Patients will be reassessed using the same instruments at 12 and 24 months after baseline interviews.

#### GP audit

GPs will be sent a list of their participating patients by fax or email. They will be asked to confirm each patient's eligibility for the study and to provide their clinical judgement in relation to each patient's dementia status using one of four options: No Dementia, Possible Dementia, Probable Dementia or Definite Dementia. GPs will be asked to complete a supplementary audit for any patients with possible, probable or definite dementia to gather data on differential diagnosis (e.g depression), memory-related tests and investigations performed (i.e. paper and pencil test; pathology; radiology) and referrals to services and specialists. GPs will return their completed audit forms to local project officer via mail, fax or email. GPs will complete audits at baseline, 12 and 24 months.

### Outcome measures

Primary outcome measures related to the first aim (collected at baseline, 12 months and 24 months) are:

1. World Health Organization Quality of Life-Bref Scale (WHOQOL-BREF) score for patients

2. Geriatric Depression Scale score for patients

Secondary outcome measures related to the first aim (collected at baseline, 12 months and 24 months) are:

1. World Health Organization Quality of Life-Bref Scale (WHOQOL BREF) for carers

2. Beck Depression Inventory score for carers

3. Agreement of GP audit for dementia with CAMCOG instrument

4. GP differential diagnosis for dementia

5. GP identification and treatment of reversible causes of dementia 6.GP dementia related referrals made

7. General Practice Assessment Questionnaire score

8. Acceptability of the process scale score

9. Services accessed

To meet the second aim of the study, screening and case finding groups (as defined above) will be compared on primary and secondary outcome measures 1 and 2 as listed above. The GPCOG score will be compared with the gold standard of the CAMCOG score to address the third aim of the study. The performance of the GPCOG against the CAMCOG will also be compared with that of the MMSE against the CAMCOG, as the MMSE is widely used to screen for dementia.

### Adverse events

Research nurses will report any subjects that they are concerned about to their local academic GP associated with the project. The GP will decide upon a course of action, up to and including reporting the matter to the subject's own GP (with subject permission). Nurses will cease interviewing subjects who report feeling tired or uncomfortable during the interview. The subject will be given the option of completing the interview at a later time, using only the data collected to that point, or withdrawing from the study.

### Sample size

It was calculated that 45 patients with dementia in each group (Waitlist and Intervention) would give a power of 0.9 to detect a 7% difference between the change in pre and post scores on any of the four domain scales of the WHOQOL-BREF with a type 1 error of .05. The population standard deviation used is 18.5 [32] and the Pearson correlation coefficient between the pre and post scores for both groups is assumed to be 0.7, based on pilot data available from the University of Melbourne.

The hierarchical nature of the study design will probably lead to some within cluster correlations and the greatest impact of this is likely to be associated with patients clustered within a GP practice. Clustering within each GP may be discounted, as it is anticipated that most GPs will have less than five patients with dementia in this study. Assuming an intra-class correlation coefficient of 0.05 and an average cluster size of 5 patients with dementia in a practice, this gives a design effect of 1.45 and a total sample size of 54 patients per group. We will therefore allow for 56 patients with dementia in the waitlist group and 112 in the intervention group to allow for comparison of outcomes between the waitlist and intervention groups overall. This sample size also allows comparison of "case finding" and "screening" patients and their support persons within the group. It is assumed that about 50% of participants will be screening and 50% case finding. Thus, we aim to recruit 168 participants with dementia overall. Based on a dementia prevalence of approximately 10% in over 75 year old Australians [37], we aim to recruit a total of 2,000 participants.

For the GDS, following the same set of assumptions and approach as for the WHO QOL-BREF (power, type 1 error and correlations), 45 patients in both the Intervention and Waitlist groups would allow detection of an average difference in the change in pre and post scores of 0.9 points on the 15 point GDS, using a standard deviation of 2.39 [28]. The cluster effect would be the same as for the WHOQOL-BREF.

For an estimate of test re-test reliability on the GPCOG a sample size of 100 will be used to ensure the correlation is estimated to a reasonable precision. The 95% confidence interval for a Pearson correlation of 0.9 would be [0.86, 0.93] and for a correlation of 0.80 [0.73, 0.85].

### Study drop outs/withdrawals

#### Subjects

We aim to recruit 2000 subjects, based on a dementia prevalence rate of 10% in the Australian population aged 75 years and over [37]. We anticipate a 10% drop out per annum, giving us 180 subjects with dementia at the 12 month mark, and 162 at 24 months, with a small number of incident cases.

#### GPs

We do not anticipate a high drop-out rate of GPs, as the demands on GPs are not onerous. As patients are consented separately to GPs, patients can continue even if their GPs withdraw from the study.

### Statistical analyses

All surveys collected on laptop computers will be downloaded to a central database. Where necessary in the analysis, the longitudinal and hierarchical/cluster nature of the study design will be taken into account using mixed effects models and other appropriate methods (see below). The primary analysis will be by intention to treat. Analyses will address the following questions:

#### Do GPs trained in the use of a screening instrument detect dementia better?

Detection rates of GPs trained in using the GPCOG (the Intervention group) will be compared with detection rates of Waitlist GPs against the 'gold standard' of the CAMCOG.

#### Do GPs trained in screening and management distinguish dementia from other conditions, particularly depression, compared with a waitlist group?

Differential diagnoses (particularly of depression), of the Intervention group will be compared with those of the Waitlist group. Misclassification rates of the GPs in the Intervention group will be compared with those in the waitlist group.

#### Do GPs trained in screening and management adhere to the guidelines?

Outcomes measured will include identification of reversible causes of dementia, tests ordered and referral patterns to specialists, support services and Alzheimer's Australia, as suggested in the guidelines.

#### Do GPs trained in screening and management provide better health outcomes?

Outcomes of Intervention and Waitlist groups will be compared at twelve months, for both patients and their carers (quality of life, depression, satisfaction with care, services accessed)

#### Do subjects with and without dementia and support persons find the screening acceptable?

All subjects and support persons will be asked to rate the acceptability of the screening instrument and procedures. Descriptive analysis will be performed. Case finding and screening groups will be compared.

#### Do GPs find the process acceptable?

GPs will be asked to rate the acceptability of the GPCOG and the Management Guidelines. Descriptive data will be reported.

#### Does case finding or screening produce better results?

Within the Intervention group, the patient outcomes for the group with memory complaints or identified by the GP as having possible or probable dementia (case finding group) will be compared with outcomes for the group without memory complaints or identified memory problems (screening group). Acceptability of the process and satisfaction with care will be particularly examined, both from a quantitative and a qualitative point of view. The performance of the GPCOG in the case finding and screening groups will be compared. In particular positive predictive value, negative predictive value and misclassification rates will be compared. GP audits will be examined to determine how many patients in each group would have a missed diagnosis of dementia if the GPCOG had not been administered.

#### Test parameters of the GPCOG

The GPCOG will be compared with the DSM diagnosis derived from the CAMCOG as a 'gold standard', and sensitivity, specificity, positive predictive value, negative predictive value and misclassification rate will be calculated. ROC curve analysis will be employed to examine the discrimination ability of the GPCOG test (i.e. ability to detect dementia) relative to MMSE. This is important as the MMSE is now commonly used in General Practice. GPCOG test-re-test and inter-rater reliability (between the nurse and the GP) will be calculated, using the kappa coefficient and intra-class correlation coefficient.

### Missing data

In the case of data missing from the study questionnaires, the questionnaires will be handled as suggested in the literature for that questionnaire. This varies from questionnaire to questionnaire.

### Steering group

The project is being overseen by a group consisting of the chief investigators, associate investigators and the project manager. This group will meet two to three times a year by teleconference and face to face. Day to day management will be undertaken by monthly project officer teleconferences.

### Ethics

Ethics approval was sought and granted initially from the Newcastle University Human Research Ethics Committee (Approval No. H-151-1205), and following this, from the appropriate Ethics Committees at each site.

## Competing interests

CDP and HB have sat on advisory boards for Pfizer, Novartis, Janssen and Lundbeck, and been speakers sponsored by Pfizer, Novartis (HB only) and Janssen (HB only). HB has been an investigator on projects funded by Pfizer, Novartis, Janssen, Lundbeck, Lilly and Sanofi, and acted as a consultant for Merck and Baxter. Other authors have no competing interests to declare.

## Authors' contributions

CDP conceived and developed this study, drafted the manuscript and has overall management of the project. HB assisted in study design and manages operations at the Sydney site. NS assisted in study design, and manages operations at the Adelaide site. JG assisted in study design, and manages operations at the Melbourne site. JM assisted in study design. PD assisted in study design, and manages operations at the Bendigo site. PM assisted in study design and drafted the manuscript. NP developed the educational intervention, assisted in study design. GH developed the educational intervention, assisted in study design. SG assisted in study design, provided project management and drafted the manuscript. BP collected data and provided project management. KM assisted in study design, collates data, will assist with statistical analysis and drafted the manuscript. All authors read and approved the final manuscript.

## Pre-publication history

The pre-publication history for this paper can be accessed here:

http://www.biomedcentral.com/1471-2296/13/12/prepub
